# Mitochondrial Phenotypes in Genetically Diverse Neurodegenerative Diseases and Their Response to Mitofusin Activation

**DOI:** 10.3390/cells11061053

**Published:** 2022-03-21

**Authors:** Xiawei Dang, Emily K. Walton, Barbara Zablocka, Robert H. Baloh, Michael E. Shy, Gerald W. Dorn

**Affiliations:** 1 Center for Pharmacogenomics, Department of Internal Medicine, Washington University School of Medicine, 660 S. Euclid Ave, St. Louis, MO 63110, USA; x.dang@wustl.edu (X.D.); em.walton15@me.com (E.K.W.); 2 Mossakowski Medical Research Institute, Polish Academy of Sciences, 02-106 Warsaw, Poland; bzablocka@imdik.pan.pl; 3 Department of Neurology, Cedars-Sinai Medical Center, Los Angeles, CA 90048, USA; robert.baloh@csmc.edu; 4 Department of Neurology, Carver College of Medicine, University of Iowa, Iowa City, IA 52242, USA; michael-shy@uiowa.edu

**Keywords:** mitochondrial dynamics, neurodegenerative diseases, mitofusin

## Abstract

Mitochondrial fusion is essential to mitochondrial fitness and cellular health. Neurons of patients with genetic neurodegenerative diseases often exhibit mitochondrial fragmentation, reflecting an imbalance in mitochondrial fusion and fission (mitochondrial dysdynamism). Charcot–Marie–Tooth (CMT) disease type 2A is the prototypical disorder of impaired mitochondrial fusion caused by mutations in the fusion protein mitofusin (MFN)2. Yet, cultured CMT2A patient fibroblast mitochondria are often reported as morphologically normal. Metabolic stress might evoke pathological mitochondrial phenotypes in cultured patient fibroblasts, providing a platform for the pre-clinical individualized evaluation of investigational therapeutics. Here, substitution of galactose for glucose in culture media was used to redirect CMT2A patient fibroblasts (MFN2 T105M, R274W, H361Y, R364W) from glycolytic metabolism to mitochondrial oxidative phosphorylation, which provoked characteristic mitochondrial fragmentation and depolarization and induced a distinct transcriptional signature. Pharmacological MFN activation of metabolically reprogrammed fibroblasts partially reversed the mitochondrial abnormalities in CMT2A and CMT1 and a subset of Parkinson’s and Alzheimer’s disease patients, implicating addressable mitochondrial dysdynamism in these illnesses.

## 1. Introduction

Mitochondria are descendants of bacterial endosymbionts that took up residence in primitive unicellular organisms approximately 1.5 billion years ago [1]. As such, mitochondria retain some characteristics of their bacterial ancestors, including partial genomes (mtDNA) and processes that repair and replicate those genomes. Mitochondrial genome repair relies heavily upon the intermixing of mtDNA and other contents from different individual organelles through mitochondrial fusion [2]. Mitochondrial quality control and replication require the reciprocal response, mitochondrial fission [3]. An imbalance between mitochondrial fusion and fission (dysdynamism), typically favoring fission and therefore provoking “mitochondrial fragmentation”, is a common feature of many neurodegenerative diseases. It is postulated that mitochondrial dysdynamism plays causal or contributory roles in such conditions [4,5].

Mitochondrial fusion and fission are mediated by dynamin family GTPases. Dynamin-related protein 1 (DRP1) is the major effector of mitochondria fission [6]; DRP1 mutations are a cause of rare severe neurological disease [7,8]. Mitofusins (MFN) 1 and 2 and Optic atrophy 1 (OPA1) are the effectors of mitochondrial fusion. MFNs mediate the initial steps of outer membrane tethering and fusion, whereas OPA1 is the effector of subsequent inner membrane fusion [2]. OPA1 mutations can cause dominant optic atrophy [9,10], while many MFN2 mutations cause the peripheral sensory–motor neuropathy Charcot–Marie–Tooth disease type 2A (CMT2A) [7,8,11].

It has frequently been observed that neurons, but not fibroblasts, from patients with CMT2A and other neurodegenerative diseases exhibit mitochondrial fragmentation, leading to the hypothesis that fibroblasts are resistant, and neurons especially sensitive, to mitochondrial dysdynamism [12,13,14]. Here, we examined the counterhypothesis that patient fibroblasts do not exhibit disease-related mitochondrial phenotypes because they are metabolically quiescent under typical culture conditions. After inducing mitochondrial-dependent respiration through substrate switching, mitochondrial fragmentation or depolarization was observed in some, but not all, of several representative genetic and etiologically diverse neurodegenerative diseases. These results suggest an individualized approach to the evaluation of mitochondrial pathology and responsiveness in preclinical experimental systems.

## 2. Materials and Methods

*Primary human fibroblasts* from CMT2A patients (*MFN2* Thr105Met and *MFN2* His361Tyr) were obtained from Dr. Robert Baloh (Department of Neurology, Cedars-Sinai Medical Center, Los Angeles, CA USA), *MFN2* Arg364Trp were from Dr. Michael Shy (Department of Neurology, University of Iowa), and *MFN2* Arg274Trp were from Dr. Barbara Zablocka (Mossakowski Medical Research Institute, Polish Academy of Sciences, Warsaw, Poland). Primary human fibroblasts from Parkinson disease (*PARK2* Arg275Trp: ND29969, *PINK1* Ile368Asn: ND40066, *LRRK2* Gly2019Ser: ND33879), Alzheimer’s disease (PSEN1 Met146Ile: ND34732, Glu184Asp: ND34730, Pro264Leu: ND34733), and normal individuals (Normal 1: ND34769, Normal 2: ND36320, Normal 3: ND29510) were purchased from the NINDS Human Cell and Data Repository. CMT1A *PMP22* DUP patient fibroblasts (ID: GM05167) were purchased from the Coriell Institute, USA. Human fibroblasts were maintained in DMEM supplemented with 15% (*v*/*v*) FBS (Gibco, Cat#: A4766801) and 1% (*v*/*v*) penicillin/streptomycin (Gibco, Cat#: 15140122) at 37 °C in 5% CO_2_–95% air. Prior to live-cell studies, fibroblasts were plated on glass coverslips and transitioned to no-glucose DMEM from Thermo Fisher Scientific (Waltham, Cat#: A1443001) with 4.5 g/L galactose (Sigma, Cat#: SLBP0699V), 1% FBS, 5 mM sodium pyruvate from Thermo Fisher Scientific (Waltham, Cat#: 11360-070) and 2 mM L-glutamine from Corning (Corning, Cat#: 34717007) and treated with Chimera (100 nM/24 h) or DMSO for 48 h [15].

*Live-cell studies assessing mitochondrial morphology and polarization status* were performed essentially as described [14]. Human fibroblasts were triple-stained with MitoTracker Green (200 nM, Invitrogen, Thermo Fisher Scientific, Cat#: M7514) to visualize mitochondria, tetramethyl rhodamine ethyl ester (TMRE, 200 nM, Invitrogen, Thermo Fisher Scientific Cat#: T-669) that labels mitochondria with intact polarization of the mitochondrial inner membrane), and Hoechst (10 ug/mL, Invitrogen, Thermo Fisher Scientific, Cat#: H3570) that stains nuclei in blue. Images were acquired at room temperature on a Nikon Ti Confocal microscope using a 60 × 1.3 NA oil-immersion objective, in HEPES-buffered Krebs–Henseleit solution (138 mM NaCl, 3.7 mM KCl, 1.2 mM KH_2_PO_4_, 1 mM CaCl_2_, 15 mM glucose, 20 mM HEPES, pH: 7.4). Laser excitation was at 488 nm with emission at 510 nm for MitoTracker Green, 549 nm with emission at 590 nm for TMRE, and 306 nm with emission 405 nm for Hoechst. Images were analyzed using Image J. 

*Fusogenicity* was quantified as an increase in mitochondrial aspect ratio (length/width). Mitochondrial dimensions from MitoTracker Green-stained confocal micrographs were measured using the Image J freehand line measurement tool [14]. Approximately 30 mitochondria per cell were measured in 12–15 cells in each independent experiment. 

*Mitochondrial depolarization* (%) was determined as the number of depolarized (negative red TMRE-stained) mitochondria relative to the total number of mitochondria (MitoTracker Green-stained) in each cell. Since red TMRE overlayed with MitoTracker Green mitochondria appears yellow in merged images, yellow mitochondria are fully polarized, and green mitochondria are depolarized [14]. Over 100 mitochondria per cell were counted in MitoTracker Green and TMRE merged images using the Image J multi-point tool. Approximately 12–15 cells were measured in each experiment, and the results were averaged to represent a single experimental n.

*mRNA sequencing library preparation and analysis* used total RNA from human fibroblasts, isolated with TRIzol Reagent (Ambion RNA Life technologies, Cat#: 15596-026,) and treated with DNase I (Invitrogen, Cat#: 18068015). mRNA sequencing was performed at the Genome Technology Access Center (GTAC) Washington University St. Louis, MO (USA) using the RiboErase protocol, with indexing and pooled sequencing on a NovaSeq S4. Sequencing depth was 30M reads per sample. RNA-seq reads were aligned to the Homo sapiens-hg19_chr22 assembly with STAR. Refseq was used as the annotation model to quantify gene counts and then normalized to CPM (counts per million). Pair-wise comparisons between transcriptomic profiles from each experimental group were analyzed using the GSA method in Partek Flow and filtered a priori (CPM (Counts per Million) ≥ 10 for at least one biological sample, *p* value < 0.05, fold change > (±) 1.5). Unsupervised hierarchical clustering using Euclidean distance and average distance was performed using Partek Flow. Pathway analyses used Database for Annotation, Visualization and Integrated Discovery (DAVID v6.8). The GEO accession number is GSE158650.

*Statistical analysis and data reporting* used one- or two-way analysis of variance (ANOVA) for multi-group comparisons. The t-test was used for comparisons of two groups; *p* < 0.05 was considered as significant. Data are reported as independent experiments, each averaging results of 8–15 cells, expressed as mean ± standard error.

## 3. Results

### 3.1. Substrate Switching Induces Mitochondrial Pathology in Cultured CMT2A Patient Fibroblasts 

CMT2A is a progressive, largely pediatric non-demyelinating peripheral sensory–motor neuropathy caused by genetic mutations in the mitochondrial fusion protein mitofusin (MFN) 2 [7,8,11]. Neuronal die-back from chronic mitochondrial dysfunction evokes neurogenic muscular atrophy of the distal upper and lower extremities. Mitochondrial fragmentation from defective fusion is one of the prototypical features of CMT2A neurons and is recapitulated in fibroblasts derived from mice with genetic ablation of MFN2 or MFN1 [12,13,14]. However, the published descriptions of mitochondrial phenotypes in CMT2A patient dermal fibroblasts are inconsistent [16,17]. Metabolic substrate switching that forces fibroblasts to use mitochondrial oxidative phosphorylation rather than non-mitochondrial glycolysis [18,19] previously induced mitochondrial shortening and other phenotypes in fibroblasts from a CMT2A patient carrying the *MFN2* R274W mutation [20]. Therefore, we generalized this approach and examined the effects of substrate switching on four different CMT2A mutant cell lines.

**Figure 1:**
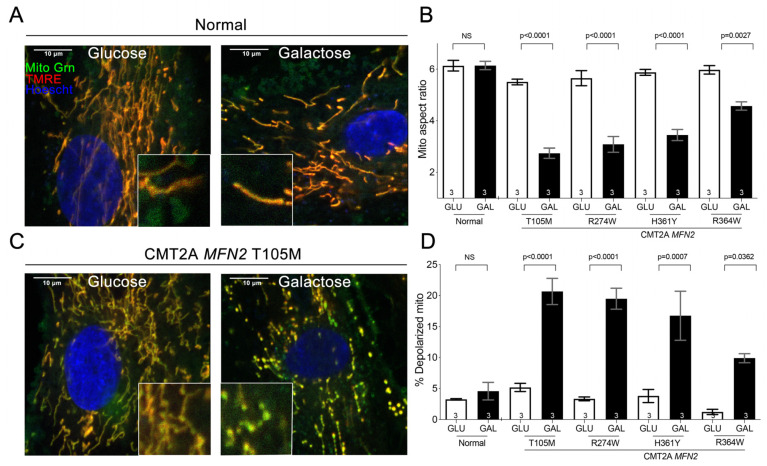
*Redirecting fibroblast metabolism with galactose provokes mitochondrial pathology in CMT2A.* (**A**,**C**). Representative confocal images of normal (**A**) and CMT2A (*MFN2* T105M) patient (**C**) fibroblasts cultured in glucose or galactose; Mito Tracker Green (green), TMRE (red), Hoechst (blue). Insets show images further magnified for clarity. Healthy mitochondria are yellow/orange; depolarized mitochondria are green. Scale bars are 10 microns. (**B**,**D**). Quantitative group data for normal and CMT2A patient fibroblast mitochondrial aspect ratio (**B**) and depolarization (**D**). Data are means ± SEM; statistics by two-way ANOVA, *p* values are at the top of the bars. NS = Nonsignificant. The number of independent experiments is shown on the bottom of each bar.

**Figure 2:**
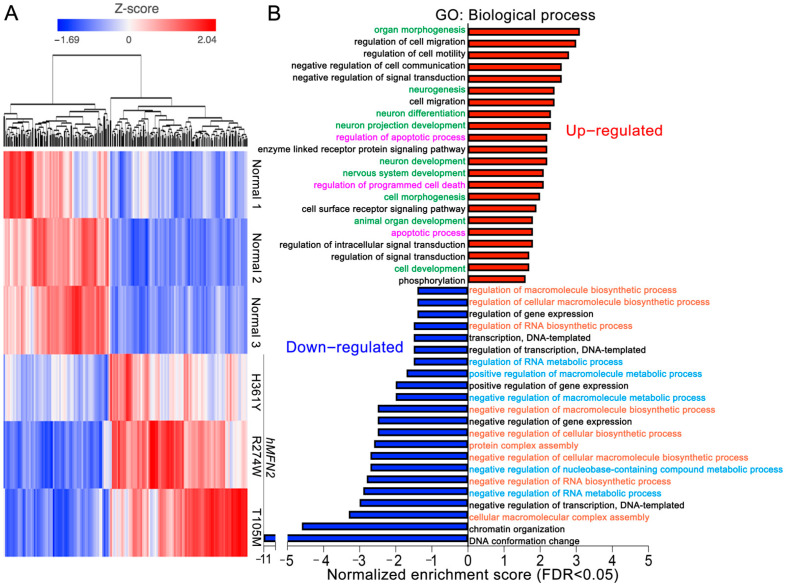
*CMT2A patient fibroblasts have a distinct transcriptional signature.* (**A**) Heat map results for unsupervised hierarchical clustering of 613 regulated mRNAs; red indicates increased expression, blue indicates decreased expression. mRNA counts in CMT2A vs. normal cells were pre-filtered for CPM ≥ 10 in at least one sample, fold change > (±) 1.5, *p* < 0.05. (**B**) Gene ontology analysis of the top 100 up- (red) and down- (blue) regulated mRNAs. Same color text indicates common pathways.

Cultured dermal fibroblasts from a normal subject had normal mitochondrial morphology and polarization status, neither of which were affected by glucose or galactose substrate present in the culture medium (Figure 1A,B,D). Likewise, fibroblasts from CMT2A *MFN2* T105M, R274W, H361Y, and R364W mutation patients cultured under standard conditions with glucose exhibited normal mitochondrial morphology and polarization status (Figure 1B–D). Strikingly, replacing glucose that is readily metabolized by mitochondrial-independent glycolysis, with galactose, which is not a facile substrate for glycolysis but is used by mitochondria for oxidative phosphorylation [18,19], provoked characteristic mitochondrial fragmentation and dissipation of the mitochondrial inner membrane electrochemical gradient, Δψm (Figure 1C,D) [12]. Moreover, as depicted in Figure 2, CMT2A fibroblasts cultured in galactose developed a distinct transcriptional signature characterized by increased levels of mRNAs linked to neuron development (green) and programmed cell death (purple), and decreased levels of mRNAs involved in metabolic (blue) and biosynthetic (orange) processes. Thus, forcing mitochondrial respiration by metabolic substrate switching induced pathological morphological, functional, and transcriptional phenotypes in multiple CMT2A MFN2 mutant fibroblasts.

### 3.2. Mitofusin Activation Corrects Mitochondrial Pathology in CMT2A Patient Fibroblasts 

**Figure 3:**
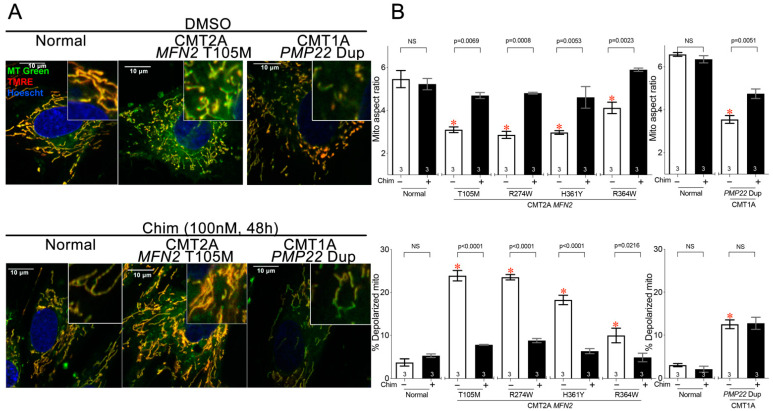
Mitofusin activation improves mitochondrial dysmorphology and dysfunction in CMT2A and CMT1A patient dermal fibroblasts. (**A**) Representative confocal images of normal, CMT2A (MFN2 T105M mutation), and CMT1A (PMP22 Dup) patient fibroblasts cultured in galactose medium; Mito Tracker Green (green), TMRE (red), Hoechst (blue). Insets show further magnified images for clarity. Healthy mitochondria are yellow/orange; depolarized mitochondria are green. Scale bars are 10 microns. (**B**) Quantitative group data for mitochondrial aspect ratio (above) and depolarization (below) from two normal patients, four CMT2A patients with different MFN2 mutations, and one CMT1A patient with PMP22 duplication. Note that the MFN2 R364W mutation causes a less severe mitochondrial phenotype. White bars indicate vehicle-treated cells and black bars indicate treatment with 100 nM Chimera for 48 h. Data are means ± SEM; *p* values from two-way ANOVA; * = *p* < 0.05 vs. DMSO-treated normal fibroblasts; individual *p* values are given for Chimera vs. vehicle within each genotype. NS = Nonsignificant. The number of independent experiments is shown on the bottom of each bar.

Since we found that CMT2A-associated mitochondrial phenotypes could be provoked in cultured patient fibroblasts, we asked if this experimental platform could be used to test the potential efficacy of an investigative therapeutic strategy through in vitro phenotype reversal. Chimera is a prototype small-molecule mitofusin activator that reportedly improved mitochondrial dysmorphology and dysfunction produced by *Mfn* gene ablation in mouse fibroblasts [14,21]. Here, Chimera (100 nM, 48 h) reversed mitochondrial fragmentation and depolarization in four genetically distinct metabolically stressed CMT2A patient fibroblast lines (Figure 3A,B, left graph).

CMT type 1 is an adult peripheral neuropathy commonly caused by duplication mutations in the myelin-associated *PMP22* gene. CMT1 is a demyelinating disease of neuron-associated Schwann cells [22], which contrasts with CMT2A that is a primary disease of neuronal mitochondria. The mechanistic basis for mitochondrial abnormalities in CMT1 is not fully understood [23]. Here, fibroblasts from a CMT1 patient developed modest mitochondrial fragmentation after galactose substitution, which was improved by Chimera (Figure 3A,B top panels, right graph), and mild mitochondrial hypopolarization on which Chimera had no effect. (Figure 3A,B, bottom panels, right graph).

### 3.3. Mitochondrial Respiratory Dysfunction in Parkinson’s Disease PARK R275W Fibroblasts Is Improved after Mitofusin Activation

**Figure 4:**
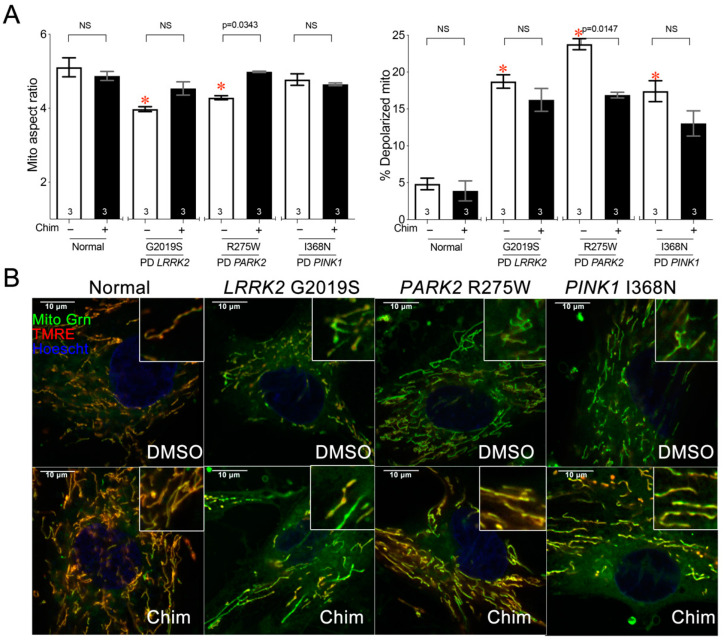
*PARK R275W Parkinson’s disease fibroblasts exhibit mitochondrial depolarization that is improved by mitofusin activation.* (**A**) Quantitative results for mitochondrial aspect ratio (left) and depolarization (right) of galactose-cultured normal and PD patients’ fibroblasts treated with 100 nM Chimera or DMSO vehicle. Data are means ± SEM; *p* values by two-way ANOVA; * = *p* < 0.05 vs. vehicle-treated normal fibroblasts; individual *p* values are given for Chimera vs. vehicle within each genotype. NS = Nonsignificant. The number of independent experiments is shown on the bottom of each bar. (**B**) Representative confocal images of studies in panel (**A**); staining and imaging as in Figure 1.

CMT2A is caused by dominant suppressive mutations in MFN2, and Chimera that improved CMT2A mitochondrial phenotypes activates mitofusins. Because the link between disease cause and therapeutic effect is direct, CMT2A may represent a “low-hanging fruit” for evaluating mitochondrial fusion/fission balance in a clinical disease. For this reason, we asked if metabolically stressed fibroblasts could also be useful for assessing mitochondrial dynamic dysfunction in a neurodegenerative disease having mitochondrial involvement not primarily caused by dysdynamism. Juvenile Parkinson’s disease is caused, in rare instances, by mutations in *PINK1* or *PARK2* (Parkin) mitophagy genes. Selective loss of dopaminergic neurons in the substantia nigra and locus coeruleus causes progressive motor dysfunction, most typically bradykinesia and resting tremor. Because mitophagy, sensed by PINK1 and effected by Parkin, is critical for mitochondrial quality control, Parkinson’s disease caused by mutations of these genes is widely considered a mitochondrial disorder [24]. Thus, we evaluated mitochondrial phenotypes in Parkinson’s disease patients’ fibroblasts carrying mutations in *PINK* (I368N), *PARK2* (R275W), and the most common Parkinson’s disease gene, *LRRK2* (G2019S). Galactose induced a modest, but statistically significant, reduction in the mitochondrial aspect ratio only in *LRRK2* and *PARK2* mutant fibroblasts (Figure 4A, left), but provoked mitochondrial depolarization in all three Parkinson’s disease cell lines (Figure 4A, right). Mitochondrial fragmentation and mitochondrial depolarization were improved by Chimera only in *PARK2* mutant cells (Figure 4A,B).

### 3.4. Mitochondrial Pathology Is Variable in Fibroblasts from Alzheimer’s Diseases Individuals 

**Figure 5:**
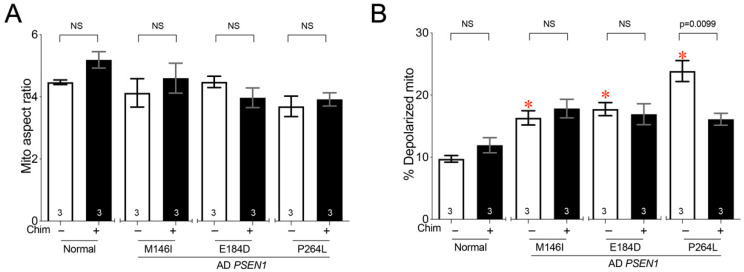
*PSEN1 mutant Alzheimer’s disease fibroblasts exhibit mitochondrial depolarization that is improved by mitofusin activation.* (**A**) Quantitative results for mitochondrial aspect ratio. (**B**) Depolarization of galactose-cultured normal and AD patients’ fibroblasts treated with 100 nM Chimera or DMSO vehicle. Data are means ± SEM; *p* values by two-way ANOVA; * = *p* < 0.05 vs. vehicle-treated normal fibroblasts; individual *p* values are given for Chimera vs. vehicle within each genotype. NS = Nonsignificant. The number of independent experiments is shown on the bottom of each bar.

Early-onset familial Alzheimer’s disease (AD) is a progressive dementia that can be caused by mutations in the presenilin (*PSEN1]* gene, among others [25]. We characterized fibroblasts from AD patients with three different *PSEN1* mutations. Although mitochondrial fragmentation has been reported in AD patient fibroblasts [25], it was not observed in our fibroblast lines under these conditions (Figure 5A, white). Moreover, mitochondrial depolarization was milder and more variable than in CMT2A and CMT1 cells, and Chimera affected only *PSEN1* P264L cells (Figure 5B, black).

## 4. Discussion

Here, we employed metabolic substrate switching to provoke mitochondrial phenotypes in primary patients’ fibroblasts representing a clinical and etiologic spectrum of rare genetic neurodegenerative diseases. We further employed this experimental platform in proof-of-concept studies evaluating whether, and in what diseases, mitofusin activation might improve disease-related mitochondrial pathologies. Mitofusin activation corrected mitochondrial dysmorphology and loss of polarization in CMT2A cells caused by *MFN2* mutations. Surprisingly, it also improved mitochondrial shortening in CMT1 cells and reversed mitochondrial fragmentation and depolarization in cells carrying a *PARK2* Parkinson’s disease mutation. Substrate switching induced mitochondrial depolarization, but not dysmorphology, in Alzheimer’s disease cells carrying three different *PSEN1* mutations, wherein mitofusin activation had no consistent effect. 

In vitro evaluation of pathological mitochondrial phenotypes in primary patients’ cells has the potential to reveal common mechanisms and predict individual therapeutic responses. Fibroblasts were the mammalian research platform of choice for foundational studies leading to the current understanding of mitochondrial dynamics [12,26,27]. The failure of most previous studies to detect characteristic mitochondrial dysmorphology and dysfunction in CMT2A patient-derived fibroblasts [16,17,28] shifted the focus of recent pre-clinical investigations to neurons in which mitochondrial dysmotility can affect cell viability. However, iPS cell-derived neurons also lose prototypical CMT2A mitochondrial phenotypes [16,29]. 

Because cultured fibroblasts are metabolically hypoactive and rely primarily upon glycolysis for energy production [19], we considered that mitochondrial metabolic activity at levels closer to those encountered in vivo might be required to manifest characteristic disease-related abnormalities. This can be accomplished by depriving cultured cells of a preferred glycolytic substrate (glucose) and substituting it with a substrate for oxidative phosphorylation (galactose) [18], as previously demonstrated in ALS [15] and CMT2A MFN2 R274W [20]. This simple maneuver induced mitochondrial fragmentation and loss of polarization in most (but not all) of the diseased cells studied and in none of the control subjects’ fibroblast lines. 

Pharmacological mitofusin activation [14] represents a new therapeutic approach that can enhance overall mitochondrial health by directly stimulating mitochondrial fusion. Chimera corrected the mitochondrial fragmentation and depolarization in CMT2A cells with four different *MFN2* mutations. The ability of Chimera to reverse mitochondrial phenotypes in CMT2A patients’ fibroblasts reportedly derives from its activation of normal endogenous MFN1 and MFN2 [14,21]. Thus, by overcoming the dominant suppression by MFN2 mutants, mitofusin activation helps to restore a more normal balance between mitochondrial fusion and fission.

All four of the CMT2A MFN2 mutant fibroblast lines studied herein exhibited mitochondrial fragmentation and dissipation of inner membrane polarization when cultured in galactose, as well as reversal with pharmacological mitofusin activation. However, in comparison to the other three CMT2A lines, these mitochondrial abnormalities were less severe in MFN2 R364W cells. The only other published functional report of this mutant concluded that it enhanced mitochondrial fusion, compared to the GTPase mutants R94Q and T105M that inhibited fusion [30]. Our data do not agree with the conclusions that MFN2 R364W is a gain-of-function mutation and that “excessive mitochondrial fusion drives CMT2A pathogenesis in a large number of patients” [30]. Indeed, these conclusions were subsequently retracted [30]. Rather, the current data in human CMT2A fibroblasts and published data in reprogrammed human motor neurons [31] lead us to conclude that MFN2 R364W is a partial loss-of-function mutant that has haploinsufficiency of MFN2 function but can nevertheless dominantly inhibit mitochondrial fusion promoted by normal MFN1 and MFN2. Importantly, the same data demonstrate unequivocally that, in the genetic context of clinical CMT2A, MFN2 R364W dysfunction is correctable by allosteric mitofusin activation. This result refutes another (retracted) conclusion in the Fissi paper: “Our data also indicate that anti-fission or pro-fusion drugs, envisioned as treatments for CMT2A or neurodegenerative disease, could be detrimental for patients with R364W and L76P alleles that would rather benefit from the development of pro-fission or anti-fusion molecules.” [30]. Here, the pro-fusion mitofusin activator corrected mitochondrial abnormalities.

We think it likely that weak intrinsic fusogenic activity of MFN2 R364W is similar to that previously described for MFN2 mutants at amino acids M376 or S378 [14]. Expression of MFN2 M376A or S378D in murine fibroblasts lacking endogenous MFN1 and MFN2 evokes mitochondrial elongation that is only about one-third as robust as wild-type MFN2 but normalizes after pharmacological mitofusin activation [14]. In the case of the M376 and S378 mutants, impairment of fusogenic activity (as opposed to complete abrogation caused by GTPase mutants) was the consequence of reduced conformational plasticity [14]. It is notable that, like MFN2 M376 and S378, MFN2 R364 is within the sequence of the MFN2-derived allosteric activating peptide designated “MP1” that binds to and interrupts the peptide–peptide interactions that critically determine MFN conformation and therefore activity [13]. Together with the current findings, this raises the possibility that MFN2 R364W may activate a similar conformational mechanism of functional impairment. It remains unclear whether actual gain-of-function (as opposed to partial loss-of-function) MFN2 mutations exist in nature and cause CMT2A. 

There are no prior data assessing whether mitofusin activation can reverse pathological mitochondrial phenotypes in conditions not caused by mitofusin defects, such as CMT1, PD, and AD. Our results show that mitofusin activation generally improves mitochondrial dysmorphology and respiratory dysfunction when there are underlying mitochondrial fragmentation and depolarization. Benefits of promoting mitochondrial fusion were observed in diseases with no known primary abnormalities of mitochondrial dynamics factors or even of mitochondrial proteins. Nevertheless, as reviewed in detail in the accompanying companion article [32], our findings raise the possibility that mitofusin activation and other techniques that normalize fusion–fission imbalances [15,33] can have therapeutic value beyond primary disorders of mitochondrial dysdynamism. 

## Data Availability

The data presented in this study are available on request from the corresponding author.
